# Experiences in Tick Control by Acaricide in the Traditional Cattle Sector in Zambia and Burkina Faso: Possible Environmental and Public Health Implications

**DOI:** 10.3389/fpubh.2016.00239

**Published:** 2016-11-09

**Authors:** Daniele De Meneghi, Frédéric Stachurski, Hassane Adakal

**Affiliations:** ^1^Department of Veterinary Sciences, University of Turin, Grugliasco-Turin, Italy; ^2^WHO Collaborating Centre for Research and Training in Veterinary Public Health, ISS-Rome, Rome, Italy; ^3^Animal Health Programme in the Republic of Zambia, Corridor Disease Control Unit, Veterinary Research Station, Mazabuka, Zambia; ^4^CIRAD, UMR CMAEE, Montpellier, France; ^5^Département des Sciences et Techniques de l’Elevage (DSTE/FASE), Université Dan Dicko Dankoulodo, Maradi, Niger; ^6^Unité URBIO, Centre International de Recherche-Développement sur l’Elevage en zone Subhumide (CIRDES), Bobo-Dioulasso, Burkina Faso

**Keywords:** tick control, acaricides, public health, environmental impact

## Abstract

Livestock, especially cattle, play a paramount role in agriculture production systems, particularly in poor countries throughout the world. Ticks and tick-borne diseases (TBDs) have an important impact on livestock and agriculture production in sub-Saharan Africa. The authors review the most common methods used for the control of ticks and TBDs. Special emphasis is given to the direct application of acaricides to the host animals. The possible environmental and public health adverse effects (i.e., risks for the workers, residues in the environment and in food products of animal origin) are mentioned. The authors present two case studies, describing different field experiences in controlling ticks in two African countries. In Zambia (Southern Africa), a strategic dipping regime was used to control *Rhipicephalus appendiculatus* ticks, vectors of theileriosis, a deadly disease affecting cattle in the traditional livestock sector in Southern Province. The dipping regime adopted allowed to reduce the tick challenge and cattle mortally rate and, at the same time, to employ less acaricide as compared to the intensive dipping used so far, without disrupting the building-up of enzootic stability. In Burkina Faso (West Africa), where dipping was never used for tick control, an acaricide footbath was employed as an alternative method to the traditional technique used locally (portable manual sprayers). This was developed from field observations on the invasion/attachment process of the *Amblyomma variegatum* ticks – vector of cowdriosis – on the animal hosts, leading to a control method aimed to kill ticks temporarily attached to the interdigital areas before their permanent attachment to the predilection sites. This innovative method has been overall accepted by the local farmers. It has the advantage of greatly reducing costs of treatments and has a minimal environmental impact, making footbath a sustainable and replicable method, adoptable also in other West African countries. Although the two methods described, developed in very different contexts, are not comparable – if public health and environmental implications are taken into account, if a balance among efficacy of the control method(s), cost-effectiveness and sustainability is reached – a way forward for the implementation of a One Health strategy can be set.

## Introduction

Livestock play an essential contribution to the livelihood of agriculture-based societies throughout the poor countries of the world (1). The sustainable livelihoods framework places great emphasis on five capital assets as a source of livelihood (namely natural, social, human, physical, and financial capital). Besides the livelihoods of the livestock owners, livestock contribute to hired caretakers, vendors, workers in related industries, as well as the consumers. Livestock, especially cattle, play a paramount role within smallholder dairy, crop-livestock and livestock-dependent systems, especially in poor countries. Most – if not all – of these production systems are at risk from ticks and tick-borne diseases (TBDs). Loss of an animal or reduction of its productivity can, in turn, affect more than one type of capital assets (1).

Tick-borne diseases affect 80% of the world’s cattle population and are widely distributed throughout the continents, particularly in the tropics and subtropics (2). It is in fact widely accepted that tick-borne haemoparasitic diseases are – and will likely continue to be – among the most important cattle diseases in the world, with higher impact in tropical and sub-tropical countries. It has been estimated that the annual global costs associated with ticks and TBDs in cattle amounted to between US$ 13.9 billion and US$ 18.7 billion (2). Ticks and TBDs represent an important proportion of all animal diseases affecting the livelihood of poor farmers in tropical countries (1). This is particularly true in Africa, where other serious vector-borne diseases (e.g., tsetse-transmitted trypanosomiasis, Rift valley Fever, etc.) occur in the same areas where the livestock population is already heavily affected by ticks and TBDs.

Briefly, the complex of vector-borne diseases, and in particular TBDs, constrains directly or indirectly the improvement and the growth of the whole livestock industry in Africa, which is of fundamental importance to rural people, by sustaining not only their food supply but also their daily income and other agricultural activities (1). More precisely, the epidemiological pattern and the risks are different according to the geographical areas (3): (i) in East, Central, and Southern Africa, where theileriosis due to *Theileria parva* is present (see below) and where European settlers introduced European cattle breeds, tick control measures have been implemented since the beginning of the twentieth century by the authorities. Thousands of dip-tanks (DTs) were thus built and cattle were regularly treated to prevent diseases transmission; (ii) in Western Africa, European farmers never introduced cattle breeds highly susceptible to the TBDs present in the areas. Local cattle did not suffer high losses due to these diseases, tsetse and trypanosomiasis being by far more prominent. Neither regional nor national tick control programmes were implemented (3). However, as the main tick species present in Central and Western Africa is *Amblyomma variegatum*, which is responsible for important direct losses, farmers were used to limiting cattle infestation by manual removal, and more recently by use of acaricide chemicals.

Ticks are thus responsible for indirect losses due to TBDs (reduction of production and mortality) but also for direct losses caused by their attachment to animal hides and blood sucking activity, leading sometimes to wounds, udder damages, weakness, and death of calves insufficiently fed by infested dams (4). Some particular tick species are also responsible for paralysis or “sweating sickness” due to the injection of toxins (1, 2).

According to Minjauw and McLeod (1), modified from McCosker (5), the major TBDs or TBDs complexes, which have a particularly severe effect on cattle, can be classified into four groups according to the tick vector species:

- *Boophilus*, now *Rhipicephalus* (*Boophilus*) spp. are vectors of species of *Babesia* and *Anaplasma* (responsible of the so-called babesiosis–anaplasmosis complex). Worldwide, anaplasmosis and babesiosis constitute the most widely distributed TBDs, having a particularly severe effect on imported (exotic) high-grade dairy and beef cattle. In 2005, the so-called blue tick, *Rhipicephalus microplus*, was introduced in Ivory Coast and Benin through the importation of cattle from Brazil (6). It is now recognized that the tick has colonized the sub-region, including neighboring countries, such as Burkina Faso, Togo, and Mali (7), inducing interactions between native and invasive cattle ticks species which have been recently studied (8). Since its introduction in West Africa, *R. microplus*, which is known to be the main vector of *Babesia bovis*, has become a major problem in traditional farms because the introduced tick populations are suspected to be resistant to acaricides, even to those of the last generations (see below).
- *Hyalomma* spp. are responsible for the transmission of the protozoan *Theileria annulata* which causes tropical (or Mediterranean) theileriosis. It occurs mainly in areas beyond the geographical regions concerned by this review (i.e., Maghreb region and Asia), where it mainly affects exotic cross-bred animals belonging to smallholders and peri-urban dairy producers. Local cattle breeds and buffalo are much more resistant.
- *Amblyomma* spp. are responsible for the transmission of the rickettsia *Ehrlichia* (*Cowdria*) *ruminantium*, which causes heartwater, a fatal disease which affects mainly sheep and goats, but also exotic cattle throughout sub-Saharan Africa. *Amblyomma* spp. also transmit the protozoan *Theileria mutans*, which causes a mild theileriosis, and it is responsible (adult *A. variegatum* ticks) for the worsening of cutaneous lesions due to the ubiquitous actinomycete *Dermatophilus congolensis*, which causes significant losses in West and Central-Southern Africa (9–11).
- *Rhipicephalus* spp. (in particular the *Rhipicephalus appendiculatus–zambesiensis* complex) are responsible for transmitting the protozoan *T. parva* which causes East Coast fever (ECF), a devastating disease in 11 countries of Eastern, Central, and Southern Africa, responsible for major losses in both small- and large-scale production systems. For more detailed information on this deadly disease, it is suggested to consult the comprehensive work by Norval et al. (12).

According to Walker (13), “*the acaricidal treatment of livestock remains the most conveniently effective way to reduce production losses from tick parasitosis and tick-borne pathogens, despite repeated predictions over many decades that this is an unsustainable*” method. This statement should however take into account the conclusions of the FAO expert consultation held in Rome in 1989 which indicated that TBDs control should be based on enzootic stability which means that in the majority of the traditional systems, particularly in areas where *T. parva* is absent, very little or even nothing needs to be done to control ticks (14). Enzootic stability, initially mentioned by Mahoney and Ross (15) to describe the epidemiology of babesiosis in Australia, is defined as the condition where the infection of all animals occurs within the period during which young calves are protected by various mechanisms (passively acquired and non-specific factors). These animals can thus develop active immunity without showing symptoms of infection and are later on immune when infected again. When they, in turn, breed, such immune cows transmit passive immunity to their offspring. The maintenance of this enzootic stability is possible only when tick infestation is high enough to allow regular infection of dams and rapid infection of calves, within the early months of their life. In such a situation, tick control should take care not to disrupt the early and regular transmission of the pathogen *via* the ticks (14, 16).

The most widely used method for the effective control of ticks is the direct application of acaricides to host animals by using the following options, as described by Minjauw and McLeod (1) and by George et al. (17):

- *dip-tanks*: dipping is an efficient, practical, and convenient mean of applying acaricide to a herd of livestock. However, it requires some permanent infrastructures to be maintained, the DT itself (with roofs, crush, and holding pens, etc.) which is expensive to build and to operate; the average capacity of a DT varies from 8,000–10,000 to 20,000–25,000 L and the amount of acaricide needed is high (generally more than 10 L of active ingredient); it requires specially trained personnel to ensure proper management (e.g., initial charging, timely and accurate replenishment of both water and acaricide, and accurate recording of the animals dipped).
  A detailed description of this method, including the design, construction, and management of DTs, is provided in the FAO field manual (18). Later in this paper, the method is described as an example of technical cooperation project for tick control by using “strategic dipping” regime (see par. Case Study 1: Field Experiences in Controlling Theileriosis by Dipping in the Traditional Livestock Sector in Southern Province of Zambia).
  An alternative method to the traditional dipping (i.e., immerging the whole animal body in a dipwash solution) was conceived in Burkina Faso from field observations carried out on the invasion/attachment process of *A. variegatum* adult ticks on cattle (19); this led to a control method aimed at killing ticks before their permanent attachment to the predilection sites using an acaricide footbath (20); it is important to note that the average capacity of a footbath is about 200 L, which is about 100 times less than a DT. Some photos and drawings on the design and construction of the footbath are provided in a technical fiche by Stachurski (21). A detailed description of this method will be given later in this paper as an example of research-development project applied to sustainable tick control [see par. Case Study 2: Footbath Acaricide Treatment, an Innovative Method to Control *Amblyomma variegatum* Ticks in the Peri-urban (Semi-Intensive) Cattle Production System in West Africa];
- *spray races*: they are more expensive and difficult to maintain than DTs as various several mechanical parts (e.g., engine, pumps, nozzles, etc.) are required, and this has restricted their use mainly to commercial farmers in most developing countries;
- *hand-spray*: it is the most widely method used by small-scale farmers for treating livestock with acaricides, but it is also potentially the least effective. As the farmers prepare and use themselves the aqueous formulation of acaricide, the concentration of the chemical may be inadequate (too low) or the amount used to treat each cattle may be insufficient (this is usually done in order to spare money);
- *pour-ons and spot-ons*: these are solutions or suspensions of acaricides to be poured along the back line of a treated animal, which spread and disperse over the whole hair/skin. These formulations are expensive, but have the advantage of not requiring water or costly equipment for their application. As the products used in pour-ons are synthetic pyrethroids (see below), they also have a long residual effect and protect animals against both ticks and biting flies. However, it should be pointed out that, sometimes, the pour-ons do not spread enough throughout the body surface to correctly control the ticks attached to the lower parts of the body;
- *hand-dressing*: this procedure involves applying acaricide to the preferred host attachment sites according to tick species (i.e., ears, udder, scrotum, perianal region, neck). As the procedure is time consuming, hand-dressing can be considered in cases where the tick burden is low and there are only a few animals to treat.

There are different classes of acaricides, among which the most commonly available and recommended (1, 17, 22) are the following:

- organophosphates (e.g., chlorphenvinphos, coumaphos, diazinon, dioxathion) and carbamates (e.g., carbaryl): these compounds are generally highly effective at low concentrations and are stable in DTs. However, organophosphates tend to accumulate in tissues or milk and are therefore not recommended for lactating cows;
- pyrethroids, mainly synthetic pyrethroids: highly effective group of acaricides that includes permethrin, decamethrin, deltamethrin, cyhalothrin, cyfluthrin and flumethrin. They typically show prolonged residual activity (at least 7–10 days) and have the additional advantage of being effective against biting flies. They are therefore used extensively in areas where trypanosomiasis is prevalent (mainly to control tsetse flies);
- amidines, which are compounds showing less prolonged residual activity (4–5 days), but no residues are found in meat or milk. The only amidine compound commercialized for tick control is amitraz.

In addition to the acaricides as such, there are also other chemical compounds to be used for tick control: macrocyclic lactones and benzoylphenylureas. The former ones (i.e., ivermectin, moxidectin, doramectin, etc.) are active against a variety of endo- and ecto-parasites besides ticks, and can be administered orally, by subcutaneous injection or pour-on application. However, these products are expensive and residues can occur in the milk and meat of treated animals for several weeks after application. The latter ones (benzoylphenylureas) are growth regulators and do not kill the ticks but disrupt their development, stopping the molting process. The best-known product, difluorobenzoylurea (Fluazuron^®^) is applied to cattle as a pour-on, acts in a systemic way but has a long residual life in tissue and milk. These products are very effective against one-host ticks such as *Rhipicephalus (Boophilus) microplus* and may be a solution where resistance to other acaricides is high (1).

Although chemicals are an important part of efforts to control ticks on cattle, they are expensive and can be detrimental to the environment and dangerous for the consumers if the recommended withdrawal periods for food of animal origins are not respected: therefore, the use of acaricides should be minimized and integrated with alternative tick control approaches (1, 2, 23). Depending on the abundance and importance of the various tick species, control strategies/treatment regimes such as seasonal treatments at the peak of tick activity (strategic or threshold tick control) or intensive dipping/spraying at the beginning of the tick season, may be sufficient to avoid economic losses due to ticks and TBDs. Effective control of TBDs is best achieved through a combination of tick control, prevention of disease through vaccination – when available – and treatment of clinical cases, where control fails (2).

Alternative non-chemical tick control methods, such as use of predators and parasites of ticks, pasture spelling (i.e., leaving pastures unstocked to break the tick’s life-cycle), anti-tick plants, tick-resistant cattle, and vaccination with tick antigens are available, but are not commonly used, and sometimes not always successfully employed (24–26).

The main methods for ticks and TBDs control are on the international research agenda for many years and have been reviewed by various authors; an integrated use of the tools available is recommended with a broader view to link TBDs control to the control of other parasitic diseases (1, 2, 26, 27).

The continuous use of chemical control to limit the harmful effects of ticks has led to the development of acaricide resistance in ticks, as it is the case with most chemicals for pest control. This is observed in particular with *R. microplus* because of the biology of this species: it is a one-host tick, accomplishing its whole parasitic cycle on the same animal within 21 days which allows the completion of 3–5 generations annually. It is therefore subject to more important selection pressure (17, 28–30). In the ‘90s, populations of *R. microplus* resistant to amidine (amitraz) were identified in Australia and South America, where ticks resistant to macrocyclic lactones were also found since 2000 (17, 30). In Africa, more precisely in Southern and Eastern Africa, one-host ticks (*R. microplus* and *Rhipicephalus decoloratus*) resistant to the majority of the different classes of “old acaricides” (but not to amitraz and macrocyclic lactones) have also been described (17, 22, 30). On the contrary, very few resistant three-host tick populations (*Amblyomma, Hyalomma, Rhipicephalus* spp. other than the former *Boophilus*) have been described in Africa (17, 30).

In West Africa, investigations carried out with *Rhipicephalus geigyi* in 2005 showed that even this one-host tick does not presently exhibit resistance to the acaricides under usage (31). At that time, acaricide resistance was not an issue of great concern; farmers continued to apply available acaricides to successfully control *A. variegatum* infestation during the main infestation period, the rainy season. Since the introduction of *R. microplus*, farmers were somewhat destabilized: in contrast to what they used to experience with *A. variegatum* infestation, *R. microplus* infest animal all along the year despite all kind of control means they may apply. Such alarming situation brought to suspect acaricide resistance in field tick populations (7). Preliminary laboratory bioassays on field tick population collected in Burkina Faso and Benin (i.e., testing almost all combinations of field isolates and acaricides) showed a strong resistance, mainly with pyrethroid such as deltamethrin and cypermethrin, in *B. microplus* populations as compared to what was observed for *Boophilus geigyi* (32).

Nowadays, the use of synthetic acaricides is still one of the primary methods of tick control, and therefore, it would be imperative to develop strategies to preserve their efficacy (30, 33). Negative aspects of the use of acaricide chemicals, besides their high direct costs, are the selection of resistant tick populations, the risk of jeopardize enzootic stability, the production losses due to handling of the animals and to the withdrawal periods, the public health implications due to toxicity, and environmental impact. In addition to that, some authors have claimed that systematic chemical control could reveal to be a non-cost-effective strategy, unless a complex set of variables (i.e., local epidemiological situation, infrastructural, and institutional constraints, etc.) are taken into account and carefully appraised (22, 34), which led some authors to suggest the strategic and threshold tick control regimes previously mentioned.

## Case Study 1: Field Experiences in Controlling Theileriosis by Dipping in the Traditional Livestock Sector in Southern Province of Zambia

The information and data reported hereunder (i.e., the set of activities described in this section: case study 1) are based on the publications, papers, and project reports by Ghirotti et al. (35); Camoni et al. (36); De Meneghi et al. (37); Scorziello et al. (38); and De Meneghi et al. (39) to which reference will be made throughout the text.

The role played by cattle in the traditional husbandry sector is of paramount importance in Zambia. National cattle herd accounts for 2.7 million head, of which 2.2 belong to the traditional agriculture system, characterized by subsistence crops, communal grazing of livestock, and cattle transhumance.

Southern province is the most important area for agriculture and livestock production in the country: it accounts for half of the national herd.

Tsetse-transmitted trypanosomiasis and theileriosis (ECF) are the two most important diseases of cattle in Zambia and constitute a major constraint to the development and productivity of the traditional cattle sector in Zambian farming.

Theileriosis had emerged as the single most important cause of mortality of cattle in Zambia: in Southern Province alone, some 30,000 head of cattle died between 1981 and 1987 (39, 40). Theileriosis due to *T. parva* is usually a fatal disease in cattle, especially in naïve adult animals and in calves. It is mainly characterized by pyrexia, lymph nodes swelling, lacrimation, anorexia and emaciation, dyspnea and pulmonary edema, digestive disturbances, abomasal ulceration, enlargement of the spleen, and lymphoid infiltration of kidneys (12).

Due to repeated outbreaks of this deadly disease and the risk of diffusion throughout the country, the Department of Veterinary and Tsetse Control Services (DVTCS), Ministry of Agriculture of Zambia, requested support and technical assistance to the Italian Ministry of Foreign Affairs, General Directorate for Development Cooperation. Hence the Animal Health Program in the Republic of Zambia (AHP), a bilateral project between the Ministry of Agriculture of Zambia and the Ministry of Foreign Affairs of Italy, was initiated and jointly implemented by the Istituto Superiore di Sanità (Higher National Health Institute of Italy) and the DVTCS. One of the main activities of the project – which started in 1987 and ended in 1992 – was the control of theileriosis in Southern Province through regular immersion of cattle in DTs containing an acaricide leading to control the main tick vector (39).

The project area included all Southern Province of Zambia, located at 25°10′–28°50′E and 15°14′–18°00′S. It covers an area of about 83,000 km^2^ and is divided in seven administrative districts. Elevation varies from 770 to 1,050 m ASL in the *valley* area, and from 1,050 to 1,400 m ASL in the *plateau* area. Annual average rainfall ranges from 500–600 mm in the *valley* to 800–900 mm in the *plateau*, with a rainfall peak in December–January. Vegetation varies in *valley* and *plateau* areas, from *mopane* to *miombo* woodland and thorny shrubs, interspersed with generally poor pasture grassland. There are three main seasons: a dry-hot period (September–November), a warm-wet period (December–April), and a cool-dry period (May–August). Climate and vegetation greatly influence the seasonality, abundance, and distribution patterns of ticks (40).

There were about 130 communal DTs distributed in the Southern province, and all operating under the AHP assistance. Farmers/livestock keepers were required to dip their animals at the DTs at predetermined intervals according to a strategic dipping regime: at 1-week interval during the high risk season (from November–December to March–April), taking into account the rain pattern and the abundance of adults ticks. From May to October, dipping was discontinued in order to allow cattle to be exposed to the mild nymphal challenge during the dry period: this allowed not completely interrupting parasite–host contacts and thus not jeopardizing the establishment of enzootic stability (39).

The acaricide, provided and distributed under the project assistance to the traditional farmers, was chlorfenvinphos, an organophosphorous compound (30% active ingredient, EC formulation). Chlorfenvinphos is a non-flammable liquid, miscible with organic solvents; it is also a lipophilic substance that may be detected in fats (e.g., milk). The degradation of chlorfenvinphos in the soil is within the range of 4–30 weeks according to the type of soil, temperature, and light. Degradation in water varies according to ph values and temperature. It is transformed in photochemical reaction. In man and animals, chlorfenvinphos is an inhibitor of cholinesterase activity, and its action occurs at both peripheral and central nervous system levels. It is toxic by inhalation, ingestion, and skin contact. Dermal exposure is the main route of pesticide absorption for workers (i.e., DTs supervisors, livestock keepers), even though inhalation is also considered important. Acute intoxication may vary from mild to severe, according to length and method of exposure and the quantity of the substance absorbed. Diagnosis of the intoxication may be difficult in mild cases when only miosis, nausea, headache, vomiting, weakness, and giddiness are observed. Severe intoxication is characterized by sudden tremors, generalized convulsions; death may occur from respiratory or cardiac failure. Chronic intoxications are rare because organophosphorus compounds are in general not highly cumulative and because, in mammals, the metabolites are usually eliminated within a few days. Nevertheless, peripheral delayed neuropathy associated with exposure to organophosphorus compounds has been observed. The severe poisoning that results from the rapid absorption of the chemical by the respiratory tract and through the skin requires that special attention is paid to protective clothing. Atropine sulphate is the antidote to be used in case of organophosphorus acute intoxication (36).

The various procedures for dipping and the general practices during DT management activities include transport, storage, mixing, and immersion of animals and final disposal of the acaricide: during these activities, vet staff and livestock owners may be at risk of exposure to harmful levels of pesticide at each stage, because of mismanagement and improper handling or accidents. An aspect which is often overlooked is the likely re-use of plastic pesticide containers by local people to store drinks and foodstuffs (36, 38).

Since acute pesticide poisoning is a serious problem in developing countries, and organophosphorus compounds seem to be one of the major causes, the AHP deemed it very important to deal with public health, occupational, and environmental health aspects related to the use of acaricide. The interventions planned and carried out by the project in the period under review were inspired by a One-Health approach *ante litteram*. Several activities aimed at preventing health and environmental hazards connected with the use of the acaricide at the DTs were planned and implemented following different phases: (i) collecting information; (ii) identifying resources; (iii) defining objectives and implementing related actions, included a feasibility study *in loco* (38).

The interventions carried out within the project framework did include an integrated set of activities which have been described in various publications, scientific papers, and project reports (35–39) to which reference can be made for more detailed information. An account of the most important and significant activities carried out within the project framework is given hereunder; data and information provided throughout this section are solely based on the papers, publications, and reports mentioned above, therefore bibliographic quotes will not be reiterated:

- assessment of the occupational hazards (i.e., ways and modalities of exposure of workers to the acaricide) as well as environmental hazards, by using an *ad hoc* questionnaire to ascertain procedures in the working environment (i.e., safety of the DTs operators, safe disposal of empty containers, accidents at work, environmental pollution, etc.);
- provision of protective equipment (e.g., plastic aprons, rubber gloves, face masks, etc.) for distribution to DTs supervisors;
- distribution of atropine sulphate vials to all the District Veterinary Offices and District Hospitals in Southern Province to be used in case of organophosphorus acute intoxication;
- training activities and conferences/seminars: on-the-job training courses/workshops on DT management, health and environmental risks related to pesticide handling were organized for all DTs supervisors and field veterinary assistants working in the project area; national seminars on DT management, ticks and theileriosis control were organized for livestock officers and veterinarians in theileriosis affected areas of Zambia;
- health promotion and health education activities: instruction leaflets on dip management (written in the local Tonga language) distributed to traditional farmers; meetings held with groups of farmers to explain the basic principles of dip management, dipping policy and the risks related to the use of acaricides; organization of a radio programme on dipping and on the related risks, broadcasted in the local Tonga language in collaboration with the National Farming Information Service (NFIS) (radio is a popular mass medium: several radio programmes in English and the major local languages are broadcasted daily all over the country, and in particular health education programmes have been developed by the Provincial Health Officers in collaboration with veterinary and agriculture extension officers); a drama group technique was used for our radio programme in order to deliver the messages in small scattered villages, as such technique is an usual communication channel in the local culture; furthermore, a TV series on agriculture (“*Lima Time*”), produced by NFIS in English language, broadcasted an episode on theileriosis and its control and prevention. Personal observations showed a good audience level and acceptability of the radio and TV messages among local people; in particular the TV programme seemed to be enthusiastically received, even though the number of TV sets is quite limited, especially in rural areas.
- Field research applied to public and environmental health: in order to investigate on the presence of acaricide residues in milk from dipped cattle under local field conditions, milk samples from traditional cattle herds were collected before dipping and at fixed intervals after dipping; in addition, samples were also obtained from the local milk collection depots; besides – as the use/re-use of empty acaricide tins was reportedly quite common in most villages of the project area – water samples stored in empty acaricide tins were collected (before and after washing with fresh water and/or with detergent) to evaluate the actual risk of re-using empty containers for storage of drinks and foodstuffs; our study demonstrated that milk collected and consumed 18–24 h after dipping appears to be safe for human consumption, according to the recommended international residues limit values, whereas acaricide residues in water stored in empty acaricide tins (although washed several times with fresh water and/or with detergent) were found to be well above the recommended safe levels, thus confirming the risks related to the re-use of plastic containers and, at the same time, providing useful information for health education activities.

To conclude, it should be stressed that although most of the risks for public health related to dipping management practices can be greatly reduced by using appropriate information/training activities, and/or by providing protective equipment, etc., there are other practices for which the impact on environmental health is not easy to prevent or to reduce significantly: for instance, when the dipwash from the DTs has to be removed at the end of the dipping season – especially if the pollution level is high (i.e., excess of dung and/or mud in the dipwash) – the option to pour it on fallow land near the DTs, to be degraded by the sunlight, is not acceptable; a solution – although not always possible and not completely safe for preventing environmental impact – could be to temporarily stock the dipwash in make shift decantation pits nearby, and then pour the dipwash on fallow land only after the active ingredient has been completely degraded by the sunlight and decanted in the pit.

## Case Study 2: Footbath Acaricide Treatment, an Innovative Method to Control *Amblyomma Variegatum* Ticks in the Peri-Urban (Semi-Intensive) Cattle Production Systems in West Africa

In Burkina Faso, as in most of Western African countries, traditional, extensive, and low-input cattle systems based on rearing of local breeds, account for most of the cattle production. The semi-intensive farming system, where exotic breeds are used to improve animal production, in particular dairy production, remains marginal: the corresponding farms, located mainly in urban and peri-urban areas, represent only 5% of the total cattle production (31). In West Africa, *A. variegatum*, more precisely the adults of this species, is the most harmful tick impairing animal growth and leading to sometimes very serious wounds (41, 42). Because the udder is one of the tick predilection sites, these wounds can result in the complete destruction of one or more teats (43). These lesions lead to an important reduction in the milk yield of dams and, consequently, to lower growth rates and higher mortality in their off-springs (4, 44). This tick exacerbates dermatophilosis cutaneous lesions (9) which are also observed on local breeds although they are less sensitive than the exotic introduced ones; besides, it transmits *Ehrlichia ruminantium*, the causative agent of cowdriosis (45). Studies have however shown that local cattle breeds benefit from a certain degree of enzootic stability to this disease, which is not the case for local small ruminants or for introduced cattle (46).

The tick control practices of traditional farmers in West Africa are thus aim mainly to limit infestation by *A. variegatum* adults, which are active during the rainy season, particularly during the first months of this period (41). Farmers in low-input systems, who used to remove these ticks manually, are now increasingly using acaricides, generally applied by manual sprayers, to control the ticks. As their income is very low, the products are frequently misused: inadequate volume is sprayed, between-treatment intervals are excessive, cheaper chemicals of uncertain origin because bought on unmonitored markets are used, and acaricides are replaced with agricultural pesticides such as those supplied for the treatment of cotton fields (20, 31).

During field studies carried out in the late ‘90s in Cameroon and Burkina Faso within the framework of research-development projects implemented by CIRAD (*Centre de Coopération Internationale en Recherche Agronomique pour le Développement*) and CIRDES, it was observed that *A. variegatum* adults do not attach to their predilection sites (udder, chests, inguinal area, etc.) as soon as they infest cattle: they first attach, not very strongly, to the interdigital areas where they remain as long as the hosts are walking and grazing (19). Ticks reach the predilection sites when the animals lie down to rest, an important proportion of them moving from one animal to another (19). As, during the rainy season, cattle are traditionally brought to graze in the savannah for about 9 h a day, they have very little time to rest or lie down (47); consequently, ticks move to the predilection sites mainly during the night and about 90% of the ticks captured on the pasture are still attached to the feet when the animals return to night paddocks in the evening (19).

Methods to treat cattle feet in order to eliminate the captured ticks and prevent them to attach to the body were looked for (43). The first attempt consisted in localized application of a flumethrin formulation at mornings, using a manual sprayer, on cattle confined in a crush-pen. The results of this trial were not optimal, important volumes of acaricide formulation being used and tick infestation on animals increasing despite treatment, partially due to the fact that ticks could move from untreated control cattle to sprayed cattle during the night, when all animals were kept together in the kraal. A footbath was then built and allowed to obtain much better results (20). Using various pyrethroids (flumethrin, alphacypermethrin and deltamethrin), cattle treatment carried out during the peak infestation period of adult ticks (i.e., from mid-May to the end of July) proved to be efficient in preventing the ticks from attaching to the predilection sites. The method was appreciated by traditional livestock farmers, essentially because it is not time consuming (once animals are familiar with the footbath, 120 animals can be treated in less than 15 min) and because it requires only about 200 mL aqueous acaricide formulation per animal at each passage, thus greatly limiting the risk of acaricide spreading in the environment. The cost of the acaricide required to treat one animal during the peak infestation period was assessed at about 130 FCFA or 0.20 €. Of course, the cost of the installation itself was not insignificant (about 330,000 FCFA or 500 €) and could not likely be afforded by a single traditional farmer. Therefore, it was suggested that the installation should be built and used by all cattle owners of a given village, more precisely by all farmers whose herds were kept for the night less than 2 km from the footbath.

Other studies showed afterward that this control method could also kill tsetse flies, at least the species present in Burkina Faso, since the legs are the most targeted parts of the body for blood meals of *Glossina tachinoides* and *Glossina palpalis* (48). Therefore, footbath treatment of cattle can not only decrease tick infestation of treated cattle but also reduce the incidence of trypanosomiasis in cattle (49, 50). Besides, as important malaria vectors, such as *Anopheles arabiensis*, feed on cattle as well as on humans and since more than 90% of these mosquitoes feed on the legs of cattle (51), such targeted cattle treatment could also have great impact on mosquito populations and contribute to malaria control of people living near cattle herds.

From 2000 to 2007, more than 50 footbaths were established in Burkina Faso, among which the majority have been installed by development projects. A few farmers even built their own footbath after noting the efficiency of the method. Experience acquired in Burkina Faso indicates that, despite scientific evidence of the efficacy of acaricide footbaths to control *A. variegatum*, a large-scale application of this tick control measure is not obvious. The acceptability of the acaricide footbath depends on farm organization and/or farming systems. Farmers working in semi-intensive and modern systems around Ouagadougou and Bobo-Dioulasso (Burkina Faso) tended to use more easily the acaricide footbath. In contrast, farmers working in the traditional husbandry sector, which is based on extensive and nomad grazing, faced some difficulties in incorporating footbath usage into their usual practices. Such difficulties persisted even for the traditional farmers that were organized within farmers’ groups (*associations d’éleveurs*). This may partly result from the paradoxical situation where, on the one hand, acaricide footbaths are necessarily fixed installations while, on the other hand, cattle transhumance is need – according the traditional husbandry system – for finding suitable pastures all year around. Moreover, it is noteworthy that any experience of tick control failure using the acaricide footbath would further enhance the unwillingness of the traditional livestock keepers to accept this tick control measure because of the efforts already experienced in adapting its use to their usual traditional practices.

A sociological study was carried out at that time in Ouagadougou and Bobo-Dioulasso to assess the adoption of this innovative tick control method (52). Authors studied the process and level of the adoption of the technology with 72 farmers. Variables describing the breeding system, the implementation and perception of the method and the knowledge of the epidemiological system were used to discriminate three clusters of farmers that were then compared using indicators of adoption. The first cluster corresponded to “modern” farmers who adopted the technique very well. The more traditional herders were discriminated into two clusters, one of which had a good adoption level, whereas the second failed to adopt the method. The economic benefit and the farmers’ knowledge appeared to have a low impact on the adoption level, whereas some modern practices (cattle breed, regular use of metallic pen, number of individual facilities) as well as social parameters (individual/collective management, kind of socio-technical network) appeared determinant. The level of technical support had also a great influence on the adoption level. What can be learned from this study is that farmers in basically traditional systems with herds in movement during wet season are not suitable for footbath implementation. However, it is expected that good results can be achieved with groups of farmers engaged in innovation (semi-intensive peri-urban production systems) with good leadership.

## Conclusion and Discussion

As it has been pointed out in the Section “Introduction,” the control of ticks and the diseases they transmit is a very complex issue. A single solution does not exist: different livestock production systems, multifaceted epidemiological patterns and diverse socioeconomic contexts are only some of the many aspects to be taken into account when tackling one of the most important constraints for animal health and production, especially in the so-called developing countries. Over the decades, the initial approach of the most widely used method for tick control – chemical treatment – significantly changed: from intensive acaricide control, aimed to “eradicate” the ticks, it was changed to more ecologically and economically sustainable acaricide control methods, such as strategic, threshold regimes. Actually, the need to reduce the costs for ticks and TBDs control and to avoid the development of acaricide resistance, and – at the same time – the consciousness and willingness to limit possible public health risks, has progressively induced the veterinary authorities, researchers, policy makers, as well as the stakeholders – including livestock breeders – to start applying an integrated control approach/package which takes into account the different options/strategies for ticks and TBDs control (2, 23, 26).

The two first hand experiences on tick control presented here – although carried out in different periods (late ‘80s–early ‘90s in Zambia, and late ‘90s–early ‘00s in Burkina Faso) and not comparable (the two areas greatly differ from the ecological, epidemiological, geographical, and socioeconomic points of view) – are a “photograph” of two different contexts where the tick control methods and strategies implemented have in common an “embryo” of attention and awareness for the possible environmental impacts for public health risks due to the use of acaricides.

As already pointed out, although the two methods cannot be compared and analyzed by using a quantitative method, the authors attempted to attribute a qualitative/semi-quantitative score by comparing the most important and relevant *pros* and *cons* of the two methods: (i) usefulness to treat one animal or many animals/one herd; (ii) overall costs (i.e., initial investment and treatment on a yearly basis/per cattle head), hence economic sustainability; (iii) environmental and public health implications and/or hazards (i.e., risk of spilling/pouring/dispersal of acaricide, risk for the operators, residues in foods of animal origin, etc.) (see Table 1).

**Table 1:** **Comparative score attributed to the tick control methods described in case study 1 and case study 2: major advantages and disadvantages**.

	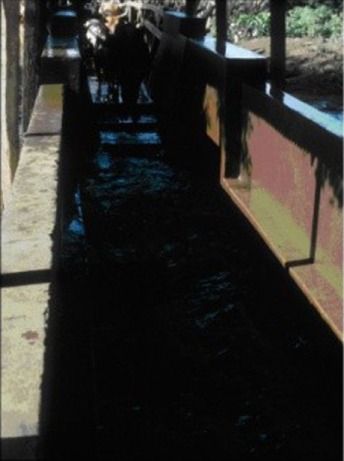	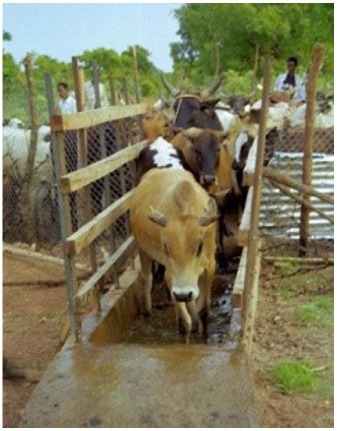	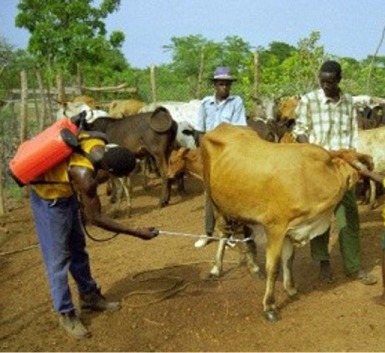	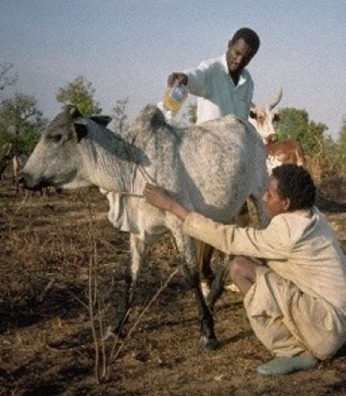

Control method	Dip-tank (case study 1)	Footbath (case study 2)	Portable manual sprayer^a^	Pour-on^a^
Initial investment	20 0000 US$	400 US$	80 US$	0 US$
Cost for the whole rainy season (per cattle head)	1.5 US$	0.2–0.25 US$	0.15–0.25 US$	3–5.5 US$
Usefulness to treat one/few animal(s)	*	*	**	***
Usefulness to treat many animals or more than one herd	***	**	*	**
Environmental implications/hazards 1. volume of product to be used	***	*	**	*
Environmental implications/hazards 2. risk of spilling/pouring/dispersal on fallow land	***	*	***	from * to *** (depending on product used)
Public health implications/hazards 1. risk for the operators	**	*	***	*
Public health implications/hazards 2. residues in foods of animal origin	***	**	*	*

*^a^Other (most) common tick control methods used under field conditions in the study areas*.

Key-legend of the score attributed: * low level; ** medium; *** high level

Unfortunately, the authors cannot report first hand any updated and follow-up information on the two projects (in Zambia and Burkina Faso) where they used to work before (both projects have been now terminated/discontinued, and no published follow-up information have been found).

As regards the case study on strategic dipping in Southern Zambia, it should be added that during the last operational period of the Italian project, a FAO project was started in Monze district (Southern province) with the aim to vaccinate cattle against theileriosis by the “infection-and-treatment” method (Muguga cocktail) (53). This was a second phase of a larger FAO regional pilot project [an earlier vaccination trial was carried out in selected areas of Southern province (54)]. The vaccination strategy required that vaccinated cattle had to be exposed to *T. parva*-infected ticks in order to allow natural post-vaccination boosters, and this created some problems/misunderstanding/lack of trustfulness in those livestock farmers who were used to apply the strategic dipping under the Italian project. Actually the “infection-and-treatment” method was a more ecologically sound method for theileriosis control as compared to dipping, and it is an important component of the so-called “integrated ticks and TBDs control package,” which was – and still is – strongly advocated and promoted internationally (2, 23, 26). When the FAO project was interrupted, the vaccination was discontinued for some years until when a new project was re-initiated under a Belgian funded technical assistance programme (a local *T. parva* stabilate/strain – not the Muguga cocktail – was then used for the infection-and-treatment vaccine) (55). Changes in theileriosis control strategies, project activities being interrupted/discontinued, intervention of different donors, and technical assistance agencies are factors which may induce cattle farmers to lose confidence in the control method(s) adopted, thus raising the need for assessing the acceptance of ECF immunization and/or other method(s) by evaluating the perception of farmers (56). The same ECF vaccination method promoted by FAO in Zambia was also used in selected cattle breeding areas in Tanzania during late ‘90s-early 2000s, under a FAO-funded project (57). Interestingly, in this case, after the FAO project assistance stopped, the vaccination was successfully continued for many years in Northern Tanzania, on a self-sustained commercial basis (58).

As regards the case study of the footbath treatment developed in Burkina Faso, after the initial demonstration of the efficacy of the method to limit *A. variegatum* adult ticks infestation, various development projects were convinced of its interest and proposed this method to farmer organizations of Burkina Faso and neighboring countries: a project, supported by CORAF/WECARD (West and Central African Council for Agricultural Research and Development) and funded by Australian CSIRO (Commonwealth Scientific and Industrial Research Organization), planned to transfer the footbath technology to other countries, targeting at first state farms in Mali (Madina Diassa) and Benin (Kpinnou) where the farmers could assess and learn the method. In Benin, where *R. microplus* tick is well established, the objective was also to check whether treatment with footbath could have any effect on *R. microplus* infestation. Unsurprisingly, the study showed that footbath treatment gave a positive result on *A. variegatum*, but was not effective against *R. microplus* because larvae of this one-host tick directly attach to the head and body of the cattle without temporary attachment to interdigital areas. As already mentioned, the socioeconomic studies carried out some few years after the introduction of this control method in Burkina Faso (52) revealed that peri-urban dairy farmers easily adopted the technique whereas traditional herders did so only if there was technical support to help them during the first months/year of use. This has to be taken into account for the potential next steps of method dissemination. On the other hand, the fact that the footbath can simultaneously reduce tick infestation and limit tsetse-transmitted trypanosomiasis (both animal and human form) could help for further acceptance of this control method. As mentioned earlier, there are a couple of examples where the treatment of cattle with insecticide/acaricide has led to indirect control effect on vectors of human diseases: in Chad, a field experience showed that treating cattle with footbath insecticide treatment has a positive effect in reducing tsetse density, hence protecting people – besides cattle – from tsetse and trypanosomes infection (50); in Ethiopia, cattle treatment with insecticide had also allowed to reduce malaria transmission by interfering with *Anopheles arabiensis* behavior and survival (51).

As a conclusion, such experiences of strategic use of acaricides/insecticides to control livestock diseases having also indirect action on vectors of human diseases are good examples of effective research-development projects whose results can be applicable at field level for integrated and sustainable disease control in poor resources countries. Once the possible public health and environmental implications of the control measures chosen have been taken into due account, and a balance has been reached among the efficacy of the control method(s), its cost-effectiveness, and sustainability, a new path can be set toward the implementation of a One Health strategy, which envisages an integrated approach for animal, human and ecosystem health.

## Author Contributions

DM: conception of the study carried out in Zambia, making analysis and interpretation of data of the study; FS and HA: conception of the study carried out in Burkina Faso, making analysis and interpretation of data of the study; DM, FS, and HA: drafting, reviewing, and finalizing the final version of the manuscript.

## Conflict of Interest Statement

The authors declare that the research was conducted in the absence of any commercial or financial relationships that could be construed as a potential conflict of interest.
